# A case of hyalinizing trabecular tumor of the thyroid: diagnostic significance of *PAX8-GLIS3* fusion

**DOI:** 10.1186/s13044-024-00196-6

**Published:** 2024-05-06

**Authors:** Shuto Hayashi, Nobuyuki Bandoh, Shogo Baba, Misaki Hayashi, Takashi Goto, Miki Takahara, Yasutaka Kato, Eriko Aimono, Hiroshi Nishihara

**Affiliations:** 1 Department of Otolaryngology-Head and Neck Surgery, Hokuto Hospital, Inadacho Kisen 7-5, Obihiro, Hokkaido 080-0833 Japan; 2 Department of Otolaryngology-Head and Neck Surgery, Asahikawa Medical University, Midorigaoka-Higashi 2-1-1-1, Asahikawa, Hokkaido 078-8510 Japan; 3 Department of Biology and Genetics, Laboratory of Cancer Medical Science, Hokuto Hospital, Inadacho Kisen 7-5, Obihiro, Hokkaido 080-0833 Japan; 4 Keio Cancer Center, Keio University School of Medicine, 35 Shinanomachi, Shinjukuku, Tokyo 160-8582 Japan

**Keywords:** Hyalinizing trabecular tumor, Thyroid gland, *PAX8-GLIS3* fusion, Papillary thyroid carcinoma

## Abstract

**Background:**

Hyalinizing trabecular tumor (HTT) is an uncommon follicular cell-derived thyroid tumor classified as a low-risk neoplasm by the World Health Organization Classification of Tumors of Endocrine Organs, 5th edition. The *PAX8-GLIS3* gene fusion is reportedly a pathognomonic genetic alteration of HTT.

**Case presentation:**

A 43-year-old Japanese female was incidentally discovered to have an 8-mm, well-defined, hypoechoic mass in the left lobe of the thyroid gland by ultrasound examination. Contrast-enhanced computed tomography scan revealed a solid mass exhibiting slight homogeneous enhancement in the lower pole of the thyroid gland. The mass was diagnosed as atypia of undetermined significance by fine-needle aspiration cytology. The patient underwent left hemithyroidectomy with routine central compartment dissection. Histologic findings revealed tumor cells with elongated nuclei and intranuclear pseudoinclusions arranged with trabeculae architecture or small nests in hyalinized stroma. Weak membranous and cytoplasmic staining was found by MIB1 (Ki-67) immunostaining. The final diagnosis was HTT of the thyroid gland. Next-generation sequencing genetic analysis of a surgical specimen revealed no pathologic mutations, including *BRAF, H/K/NRAS,* or *RET-PTC* fusions. The *PAX8-GLIS3* fusion was detected by RT-PCR.

**Conclusions:**

A rare case of HTT was demonstrated through imaging, cytologic, histologic and molecular investigations. *PAX8-GLIS3* fusion detected by RT-PCR and Sanger sequencing was confirmed to be a genetic hallmark of HTT.

**Supplementary Information:**

The online version contains supplementary material available at 10.1186/s13044-024-00196-6.

## Background

Hyalinizing trabecular tumor (HTT) is an uncommon follicular cell-derived thyroid neoplasm proposed by Carney et al. in 1987 as a subtype of follicular adenoma [1]. HTT was initially included in the World Health Organization (WHO) Classification of Tumors of Endocrine Organs in 2004. In the 5th edition of the WHO classification, published in 2022, HTT was classified as a low-risk neoplasm characterized by an excellent prognosis, although it cannot be categorized as benign [2]. Histologically, HTT is composed of large trabeculae of elongated or polygonal eosinophilic cells admixed with intratrabecular hyaline material. Cytomorphological diagnosis of HTT is challenging due to the significant overlap of nuclear features on fine-needle aspiration cytology (FNAC), such as nuclear grooves and intranuclear pseudoinclusions, similar to papillary thyroid carcinoma (PTC), which requires radical surgery [3]. Nikiforova et al. highlighted the significance of the *Paired box 8 (PAX8)-Glis family zinc finger 3* (*GLIS3)* fusion as a genetic hallmark of HTT in 2019 [4]. To date, given the rarity of HTT, only a few reports have demonstrated the *PAX8-GLIS3* fusion in HTT. We present a case of HTT in which the *PAX8-GLIS3* fusion was detected by reverse transcription (RT)-polymerase chain reaction (PCR) analysis.

## Case presentation

**Fig. 1:**
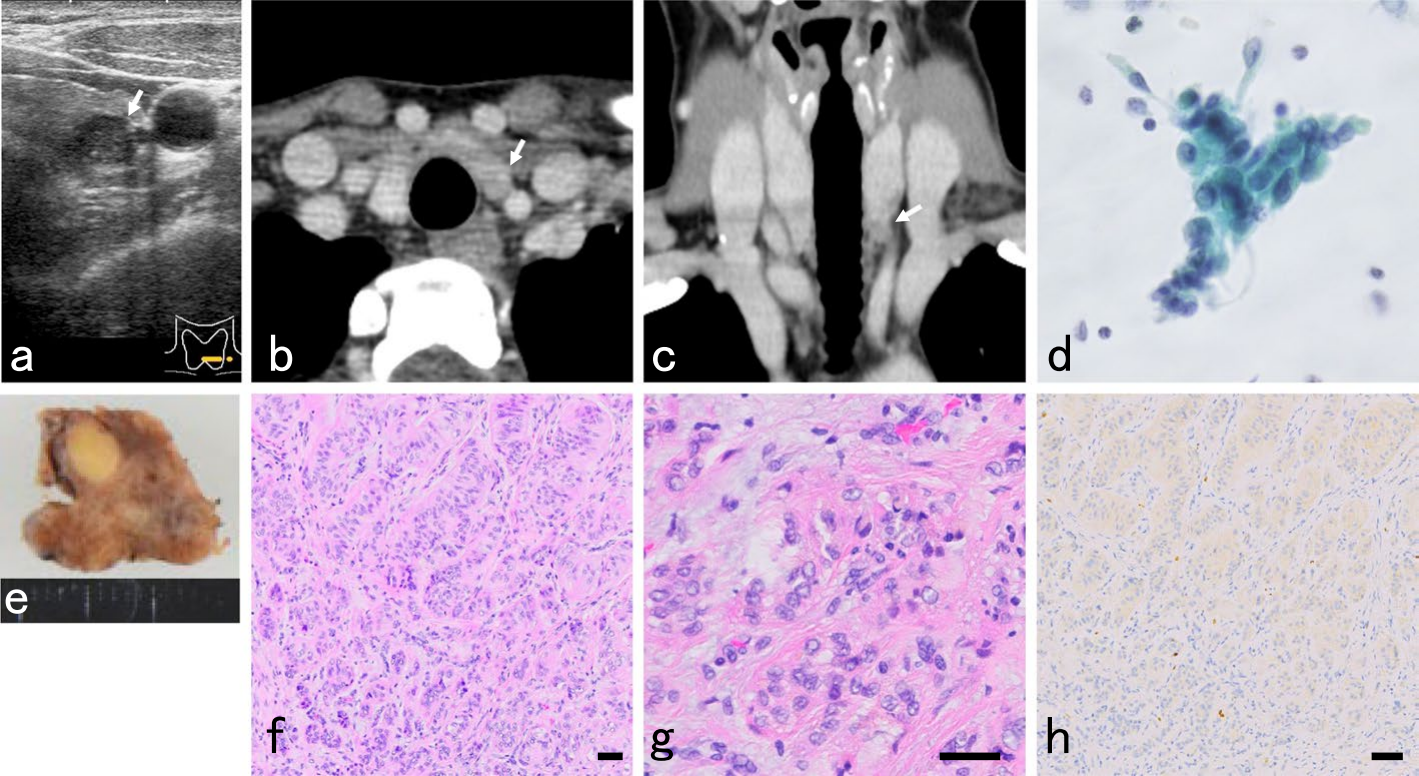
Imaging and histologic findings of the hyalinizing trabecular tumor. Ultrasonography revealed a well-defined, hypoechoic mass with microcalcification measuring 8 × 7 × 7 mm in the left lobe of the thyroid gland (**a**). CT scan revealed solid mass exhibiting slight homogeneous enhancement in the lower pole of the thyroid gland (**b**, **c**). FNAC demonstrated various clusters of follicular cells with enlarged and polygonal nuclei, fine granular chromatin, and intranuclear pseudoinclusions. Nuclear atypia was mild. The tumor was diagnosed as atypia of undetermined significance (AUS) (**d**). The specimen appeared as a well-circumscribed, tan-white, and homogeneous nodule (**e**). Histologically, the nodule consisted of wide trabeculae or small nests delimited by stromal bundles. Within the trabeculae, a variably abundant eosinophilic hyaline material was present, enveloping tumor cells. Tumor cells were large or medium-sized and polygonal or elongated, oriented perpendicular to the trabeculae (**f**). Tumor cell nuclei were convoluted with prominent membrane irregularities that formed intranuclear pseudoinclusions (**g**). Weak membranous and cytoplasmic staining was found by MIB1 (Ki-67) immunostaining (**h**). Scale bars: 50 μm

A 43-year-old Japanese female was incidentally discovered to have a well-defined, hypoechoic mass with microcalcification measuring 8 × 7 × 7 mm in the left lobe of the thyroid gland (Fig. 1a) and 3 paratracheal lymph nodes measuring 6 mm by ultrasound examination. Contrast-enhanced computed tomography (CT) scan revealed a solid mass exhibiting slight homogeneous enhancement in the lower pole of the thyroid gland (Fig. 1b, c). Serum thyroglobulin, free thyroxine, free triiodothyronine, and thyroid-stimulating hormone (TSH), and TSH receptor antibody were 26.5 ng/mL, 1.1 ng/mL, 2.4 pg/mL, 3.39 μIU/mL, and < 0.8 IU/mL, respectively, and were within normal levels. Serum anti-thyroid peroxidase, and thyroglobulin antibody levels were elevated, at 319 IU/mL and 38.8 IU/mL, respectively. FNAC demonstrated various clusters of follicular cells with enlarged and polygonal nuclei, fine granular chromatin, and intranuclear pseudoinclusions. Nuclear atypia was mild. Nuclear grooves and hyaline matrix were not seen (Fig. 1d). The tumor was diagnosed as atypia of undetermined significance (AUS) according to the Bethesda system [5].

The patient underwent left hemithyroidectomy with central compartment lymph node dissection under general anesthesia. The specimen was serially sectioned, revealing a well-circumscribed, tan-white, and homogeneous nodule (Fig. 1e). Histologic findings revealed that tumor cells were arranged in wide trabeculae or small nests delimited by stromal bundles (Fig. 1f). Within the trabeculae, a variably abundant eosinophilic hyaline material was present, enveloping the tumor cells. Tumor cells were large or medium-sized and polygonal or elongated, oriented perpendicular to the trabeculae. The tumor cell nuclei were convoluted, with prominent membrane irregularities that formed intranuclear pseudoinclusions (Fig. 1g). No tumorous lesions were found in the dissected paratracheal lymph nodes. Immunohistologic examinations were negative for carcinoembryonic antigen (CEA), cytokeratin 19, hector battifora mesothelial (HBME)-1, S-100, and 34βE12 (data not shown). Weak membranous and cytoplasmic staining was found by MIB1 (Ki-67) immunostaining (Fig. 1h). The diagnosis was made as HTT of the thyroid gland. No evidence of recurrence or metastasis has been observed as of 10 months postoperatively.

**Fig. 2:**
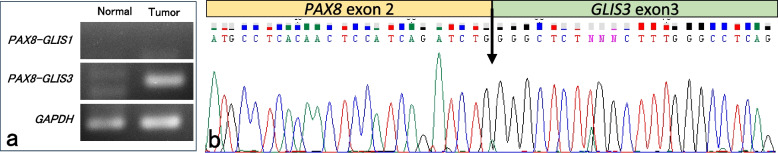
Genetic analysis by RT-PCR and Sanger sequencing. In RT-PCR analysis of the tumor, only a *PAX8-GLIS3* PCR product in the surgical specimen was observed (**a**). Sanger sequencing was used to generate an electropherogram of the genomic *PAX8-GLIS3* fusion gene and indicate the fusion point (arrow) (**b**). Images were produced using the free software Chromas version 2.6.6 (http://technelysium.com.au) and then modified

Next-generation sequencing (NGS)-based cancer panel testing was performed as previously described [6]. Briefly, total DNA was extracted from 10-μm-thick formalin-fixed paraffin-embedded (FFPE) tissue sections of the tumor specimens. Capture probes using SureSelect PrePool Custom Tier2 (Agilent Santa Clara, CA) were employed to detect mutations in the target regions of 144 caner-related genes and fusions of 11 genes (Additional file 1). The libraries were sequenced using an Illumina MiSeq platform (Illumina, San Diego, CA). No pathologic mutations were detected in *BRAF, RET, H/K/NRAS*, and the *TERT* promoter, and no *RET-PTC* fusions were detected. RT-PCR analysis to detect *PAX8-GLIS1* and *PAX8-GLIS3* fusion genes was performed as previously reported [4] and originally modified. Briefly, RNA from FFPE tissue sections from the tumor and thyroid tissue without the tumor were reverse transcribed using a NucleoSpin® total RNA FFPE XS system (Takara, Tokyo, Japan). RT-PCR analyses were conducted using a OneStep RT-PCR kit (Qiagen, Valencia, CA) and the primers reported in Additional file 2 [4]. The PCR products were purified and sent for sequencing (Azenta Life Sciences, Tokyo, Japan). A *PAX8-GLIS3* PCR product was detected in the surgical specimen (Fig. 2a). Sanger sequencing was used to generate an electropherogram of the genomic *PAX8-GLIS3* fusion and fusion point (Fig. 2b).

## Discussion and conclusions

HTT accounts for only 1% of all thyroid tumors with a female predominance and is most common in people in their 50 s [4, 7]. HTT is diagnosed based on histological findings of prominent trabecular or organoid growth patterns of elongated cells oriented perpendicular to the trabeculae with intratrabecular hyaline material [7, 8]. Neoplastic cellular proliferations are usually well-demarcated and exhibit nuclear features that overlap with PTC, such as nuclear grooves and intranuclear pseudoinclusions. In immunohistologic analysis, HTT exhibits membranous and cytoplasmic staining with MIB1, which can be diagnostic in differentiating HTT from PTC [9]. HTT is also positive for thyroglobulin and thyroid transcription factor-1 [7]. Focal and weak staining of HBME-1 and galectin-3 has also been reported. HTT is usually negative for calcitonin, CEA, chromogranin, and cytokeratins.

Cytologic diagnosis of HTT remains challenging, as it displays some cellular characteristics similar to PTC [10]. Although HTT exhibits several unique cytomorphological features, such as the presence of trabecular-like pattern, hyaline matrix, and intracytoplasmic yellow body, both HTT and PTC can exhibit hypercellularity, cellular atypia, intranuclear pseudoinclusions, and nuclear grooves on FNAC [7]. In the present case, in consideration that nuclear atypia was mild and nuclear groove was absent, the tumor was diagnosed as AUS on FNAC. In addition, the tumor was not diagnosed as HTT due to the lack of trabecular-like clusters, hyaline matrix, and yellow body. As we could not rule out the possibility of PTC with the results on FNAC and the ultrasound findings of the presence of microcalcification and paratracheal lymph nodes, hemithyroidectomy and central compartment dissection were performed.

In the present case, we used RT-PCR analysis and Sanger sequencing of a surgical specimen from a patient with HTT to clearly demonstrated the presence of a *PAX8-GLIS3* fusion. *PAX8*, located on chromosome 2q14.1, is a paired box transcription factor that is highly expressed in differentiated thyroid follicular cells and required for normal thyroid development and function [11]. *GLIS3*, located on chromosome 9p24.2, belongs to the GLI-similar zinc finger transcription factor family and can function as either an activator or repressor of gene transcription. In the thyroid, *GLIS3* is an important regulator of thyroid hormone biosynthesis [12]. The DNA-binding domain is encoded by exons 4–6 of *GLIS3*. The fusion point of the chimeric transcript of the *PAX8-GLIS3* fusion was between exon 2 of the *PAX8* gene and exon 3 of the *GLIS3* gene. Therefore, the chimeric transcripts are regulated by the *PAX8* gene promoter and preserve the zinc-finger-containing DNA-binding domains of GLIS3 [4]. The *PAX8-GLIS3* fusion leads to significant overexpression of extracellular matrix-related genes (multiple collagen genes), which is likely responsible for excessive collagen synthesis and deposition in HTT [4]. The resulting considerable hyalinization gives these tumors their distinctive microscopic appearance and nomenclature.

To date, only 2 reports have demonstrated the presence of *PAX8-GLIS3* fusions using surgical specimens from patients with HTT. Nikiforova et al. demonstrated for the first time in 2019 the *PAX8-GLIS3* fusion in 93% of 14 patients with histologically confirmed HTT and the *PAX8-GLIS1* fusion in 7% of these cases [4]. In the same year, a multi-institutional study confirmed the presence of the *PAX8-GLIS3* fusion in 8 patients with histologically verified HTT; however, no thyroid tumors of varying histology, including PTC, harbored the *PAX8-GLIS3* fusion [13]. Whole-exome sequencing, RNA sequencing, targeted NGS, fluorescence in situ hybridization, and immunohistochemistry have been used to detect *PAX8-GLIS3* fusion [4, 13]. In the present case, we detected the *PAX8-GLIS3* fusion using RT-PCR with Sanger sequencing confirmation, which is a more convenient approach than whole-exome and RNA sequencing. We also performed the NGS-based panel testing. However, the panel we used for NGS-based testing did not include the *PAX8-GLIS3* fusion. We were able to exclude the presence of mutations in *BRAF, RET, H/K/NRAS*, and the *TERT* promoter as well as *RET-PTC* fusions, which are often detected by NGS in the thyroid tumors of varying histology. A previous report of one case described the detection of the *PAX8-GLIS3* fusion by NGS genetic analysis of fine-needle aspirates from 180 thyroid nodules, leading to a diagnosis of HTT [14]. Molecular testing of FNAC samples for the markers including the *PAX8-GLIS3* fusion can dramatically enhance the precision of preoperative FNAC.

The majority of HTT are clinically benign even after long-term follow-up, and complete excision is generally curative [2]. A study demonstrated no locoregional recurrence or distant metastasis in 29 cases with 6-year follow-up after surgery [15]. However, one case of 9 tumors with HTT was reported to have a neck lymph node metastasis [16]. In a study of 119 cases with a follow-up of 13–48 months, only one case with histologic findings of capsular and vascular invasion caused lung metastasis [17]. Therefore, we consider that long-term follow-up will be needed in the present case.

In conclusion, a rare case of HTT was demonstrated by imaging, cytologic, histologic, and molecular investigations. Molecular identification of the *PAX8-GLIS3* fusion by RT-PCR with Sanger sequencing confirmation may aid in making the correct diagnosis.

### Supplementary Information

**Additional file 1.** Panel of cancer-related genes examined using next-generation sequencing.

**Additional file 2.** Primers and conditions used for RT-PCR analysis of *PAX8-GLIS1*and *PAX8-GLIS3* fusions.

## Data Availability

No datasets were generated or analysed during the current study.

## Abbreviations

BRAF: v-raf murine sarcoma viral oncogene homolog B
CEA: Carcinoembryonic antigen
FNAC: Fine-needle aspiration cytology
GLIS3: Glis family zinc finger 3
HBME-1: Hector battifora mesothelial-1
HTT: Hyalinizing trabecular tumor
NGS: Next-generation sequencing
PAX8: Paired box 8
PTC: Papillary thyroid carcinoma
RAS: Rat sarcoma virus
RET: Rearranged during transfection
RT-PCR: Reverse transcription-polymerase chain reaction
TERT: Telomerase reverse transcriptase
